# Using the Complex Network Model to Associate Nutritional, Psychological, and Physical Parameters and Aspects of Sleep with Depression Symptoms

**DOI:** 10.3390/jcm13226743

**Published:** 2024-11-09

**Authors:** Pedro Paulo Menezes Scariot, Ana Luiza Paula Garbuio, Andrea Corazzi Pelosi, Larissa Castro Pedroso, Larissa Albano Hipólito Silva, Stella Antunes Berigo, Ivan Gustavo Masselli dos Reis, Leonardo Henrique Dalcheco Messias

**Affiliations:** Research Group on Technology Applied to Exercise Physiology (GTAFE), Laboratory of Multidisciplinary Research, São Francisco University, Bragança Paulista 12916-900, SP, Brazil; pedroppms@yahoo.com.br (P.P.M.S.); anagarbuiog@hotmail.com (A.L.P.G.); andrea.corazzi@usf.edu.br (A.C.P.); larissacapedroso@gmail.com (L.C.P.); la.albano@hotmail.com (L.A.H.S.); stella.berigo@mail.usf.edu.br (S.A.B.); ivan.reis@usf.edu.br (I.G.M.d.R.)

**Keywords:** mental disorders, nutrition, physical exercise, sleep quality, cognitive dysfunction, eating habits

## Abstract

**Background/Objectives**: Major depressive disorder is a significant public health concern linked to factors such as a low-quality diet, a sedentary lifestyle, and poor sleep quality, all of which contribute to its development; nevertheless, the existing literature lacks a comprehensive framework to effectively integrate these interrelated influences. **Methods**: To address this gap, we conducted a questionnaire-based study involving 411 individuals aged 18 to 74 and employed a weighted complex network model to clarify the associations among nutritional factors, physical activity levels, psychological parameters, and sleep profiles and depression. In addition to constructing networks that encompass distinct subgroups based on general context, sex differences (female vs. male), and four age categories, our network was designed with a clearly defined target: the score from the Beck Depression Inventory. **Results**: In all networks studied, psychological parameters (e.g., tension, depression, hostility, fatigue, confusion, and total mood disturbance) emerged as the most influential nodes in relation to the targeted node (Eigenvector centrality of approximately 0.30). Additionally, sleep quality was identified as the next most relevant parameter for the general network (Eigenvector = 0.25), while nutritional factors—particularly carbohydrates—demonstrated greater prestige within the male network (Eigenvector = 0.06). Nutritional parameters had a stronger influence on depressive symptoms among individuals aged 29–39 years (Eigenvectors = 0.09, 0.09, and 0.14 for energy, carbohydrates, and fat, respectively). **Conclusions**: This novel approach allowed for a clearer visualization of how the studied parameters impact depressive symptoms, revealing significant differences when certain aspects are examined independently across distinct groups.

## 1. Introduction

Major depressive disorder is one of the world’s public health problems that has gained prominence in recent years [1]. The most frequent symptoms experienced by individuals with depression include persistent fatigue, cognitive dysfunction, alterations in appetite and libido and suicidal thoughts [2], alongside the dysregulation of psychological factors such as mood disorders [3]. Evidence indicates that lifestyle/environmental factors play a substantial role in the development of depressive symptoms [4,5]. In regard to factors, those associated with physical activity, diet, and sleep appear to be the most critical. Studies have reported that individuals with low levels of physical activity are at increased risk of developing depression [6,7,8]. A number of studies have also demonstrated that depression can be exacerbated by a high consumption of processed foods rich in refined grains, sugars, and saturated fats [9,10,11,12,13,14]. Additionally, insufficient quality and quantity of sleep can alter the neuroendocrine profile, resulting in increased cortisol and reduced serotonin, both of which are involved in the pathogenesis of depression [2,15,16,17,18,19].

Most research on depression has focused on pairwise relationships (bivariate analysis), with physical activity, dietary patterns, sleep profiles, and psychological aspects analyzed separately in relation to depressive symptoms. While these studies are valuable, they cannot definitively identify which variables are the most influential for depression. Given that the etiology of depression is inherently complex and cannot be attributed to a single factor [20,21,22], there is an urgent need to establish a more integrated model that “holistically” connects the various variables associated with depression. One promising approach to achieve this is through network analysis, and some interesting initial attempts have been made to associate variables involved in depression using this method. For example, Wang et al. [23], using the bridge centrality index, identified notable connections between depression–anxiety symptoms and lifestyle factors (tobacco use, alcohol consumption, habitual diet rhythm, physical exercise frequency). Utilizing both between-person and within-person network models, Anderson et al. [24] investigated how factors such as physical activity, sleep complaints, smoking, socioeconomic status, social interaction, and racial status relate to depression in a large sample of U.S. adults. Additionally, Ref. [25] employed network analysis on data from the Child Depression Inventory to identify central depressive symptoms across various school-aged periods. Given that this field is still in its early stages of scientific exploration, further research is essential. Specifically, comprehensive evaluations are needed that encompass a detailed mapping of macronutrient intake and thorough assessments of exercise patterns. Additionally, employing various network strategies or mathematical approaches that consider determinants such as age and sex could enhance our understanding of depression-related factors. To help address some of these gaps in the literature, we collected data on physical activity using the IPAQ, psychological parameters through the POMSs questionnaire, nutritional information via the Food Frequency Questionnaire, and sleep parameters assessed with the Pittsburgh Sleep Quality Index and the Epworth Sleepiness Scale.

We employed eigenvector centrality to associate depression-related variables, offering a perspective that distinguishes our work from existing studies. This centrality metric not only considers direct connections but also assesses the overall influence of a node’s connections within the entire network. Consequently, a variable with high eigenvector centrality is not only well connected but also linked to other influential nodes, highlighting its significance in the broader context of the network [26,27]. In other words, Eigenvector centrality is a suitable measure when it is believed that a variable’s prestige is influenced by the importance of the variables it is connected to. Moreover, in the present study, networks were constructed with a clearly defined target. We consider the score from Beck’s Depression Inventory [28,29] to be suitable because it is capable of capturing a broad spectrum of depression-related feelings in a single value. Additionally, the use of a targeted network facilitates the interpretation of relationships, providing clearer insights compared to non-targeted network analyses.

While it might seem most intuitive to conduct an analysis that incorporates all the data into a general network, we also constructed targeted networks based on datasets derived from distinct subgroups. In addition to examining sex differences (female vs. male), we stratified participants by age into four categories: G1 for those aged 18 to 28 years, G2 for those aged 29 to 39 years, G3 for individuals aged 40 to 50 years, and G4 for participants aged over 51 years. To our knowledge, this approach of constructing networks from specific population segments has not been previously applied in depression research. By adopting this strategy, unique patterns and relationships that may be obscured in a general analysis can be uncovered, ultimately enhancing the specificity and applicability of our findings. Regarding the hypotheses, a strong significance of total mood disturbance (TMD) within the network was predicted, as this variable encompasses all items from the Profile of Mood States (POMSs). Given that females are at a higher risk than males for experiencing depressive symptoms [30,31,32], we postulate that the networks of women and men will exhibit markedly distinct patterns and connectivity. Finaly, as sleep is of utmost importance for overall health [33], we expected to observe a strong relevance of sleep quality on the target.

## 2. Materials and Methods

### 2.1. Experimental Design

**Figure 1:**
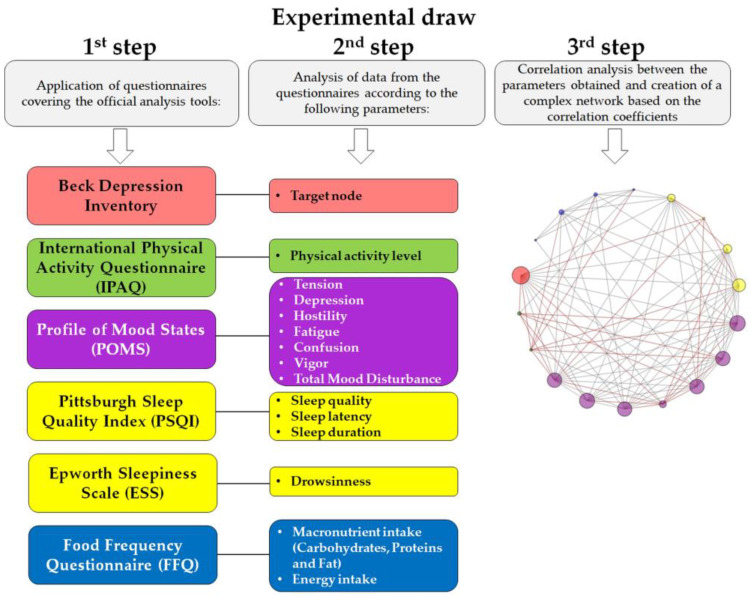
An experimental diagram illustrating all stages of the work performed, detailing each questionnaire along with select topics utilized for each parameter in the target complex network.

This experiment consists of a cross-sectional design with participants from Bragança Paulista, São Paulo, Brazil. The experiment was divided into three stages (Figure 1). After obtaining participant consent and gauging interest in the research through digital means, inclusion and exclusion criteria were established, and scales assessing depression symptoms, physical activity levels, mood states, sleep profile, and dietary recall were administered digitally. Given that data collection took place from September 2021 to August 2022, the initial phase coincided with the global COVID-19 pandemic. Therefore, in alignment with national guidelines for viral containment, we opted for digital data collection. Although many activities resumed as COVID-19 cases declined, we decided to continue digital data collection to ensure consistency in this stage. Stages 2 and 3 involved the analysis of the collected data. In Stage 2, the questionnaire results were compiled, and in Stage 3, these data were analyzed using a complex network model.

### 2.2. Participants

No pre-existing lists were used to access the sample. Recruitment was conducted via institutional outreach channels, such as the website and social media, allowing for participants to contact researchers through institutional email addresses. All procedures adhered to the guidelines outlined in Circular Letter No. 1/2021-CONEP/SECNS/MS issued by the Brazilian Ministry of Health. Next, an interview was conducted digitally to assess each volunteer’s eligibility for inclusion in the sample. Inclusion criteria were set as follows: (1) being between 18 and 74 years of age, (2) familiarity with digital questionnaires on the Google Forms platform^®^, (3) literacy, and (4) confirmation that topics associated with the questionnaire’s objectives would not cause discomfort. Conversely, individuals were not considered if (1) they were under 18 years of age or over 74; (2) were already diagnosed with depression; (3) those who did not respond to all the questionnaire items; (4) those who reported discomfort with answering specific questions; or (5) those who declined to continue participation after understanding the questionnaire details; these individuals were excluded from this study. Four hundred and eleven individuals of both sexes who answered all the questions present in the questionnaires were included. Individuals were divided into categories according to sex and age, as schematized in Table 1.

### 2.3. Procedure and Instruments

The questionnaires used in this study were administered via Google Forms. During the completion period, a member of the research group was available to answer any questions regarding the items.

#### 2.3.1. Depression Symptomatology (The Target Node in Network)

To assess the level of depression, we chose the Beck Depression Inventory, originally developed in 1961, with reference to the original instrument available here [28]. We utilized a validated questionnaire for our language [29]. The scale comprises 21 items, which assess depressive symptoms and attitudes, and each item is rated on a scale from 0 to 3, with higher scores indicating more severe symptoms. The inventory’s items aim to assess symptoms and attitudes related to sadness, pessimism, feelings of failure, lack of satisfaction, feelings of guilt, feelings of punishment, self-depreciation, self-accusations, suicidal ideation, crying/crying crises, irritability, social withdrawal, indecision, body image distortion, work inhibition, sleep disturbance, fatigability, loss of appetite, weight loss, somatic concerns, and decreased libido. The sum of all items yields a final score associated with the symptoms, serving as a prominent parameter within the network.

#### 2.3.2. Assessment of Physical Activity Level

To evaluate an individuals’ overall activity levels and habits related to inactivity, the International Physical Activity Questionnaire (IPAQ) was adopted. This questionnaire is endorsed by the World Health Organization and was developed in partnership with representatives from various countries, including Brazil [34]. The validated IPAQ has shown correlation coefficients close to 0.80, indicating good reproducibility [35]. The short form of the IPAQ we used includes 8 questions, which can be found in the native language in the final attachments of Matsudo et al. [35]. The IPAQ evaluates vigorous and moderate physical activity (for at least 10 continuous minutes) by measuring both weekly frequency and daily duration. The questionnaire guides respondents to distinguish between vigorous and moderate activities based on the intensity of sweating, breathing, and heart rate. Additionally, the IPAQ captures any form of walking for 10 min or more. Lastly, it assesses sedentary behavior by measuring the time spent sitting during workdays and weekends. The final score for this application is given in MET-minutes per week, where MET represents an estimate of metabolic equivalents.

#### 2.3.3. Assessment of Psychological Parameters

The POMSs, originally developed by McNaIr et al. [36], is a widely recognized tool for assessing mood states. We utilized a reduced version (translated into Portuguese) by Viana et al. [37], ensuring its applicability to our specific research context. This version comprises 42 items, including 5 negative domains: tension (T), depression (D), hostility (H), fatigue (F), and confusion (C), along with a positive domain, vigor (V), each containing six items. Still, through the POMSs questionnaire, it is possible to obtain the total mood disturbance (TMD). The TMD is calculated by summing the five negative scales (T + D + H + F + C), subtracting the vigor scale score, and adding a constant of 100 to prevent negative overall results.

#### 2.3.4. Assessment of Sleep Parameters

The Epworth Sleepiness Scale (ESS) was employed to evaluate daytime sleepiness. The ESS was originally proposed by Johns [38] in 1991. To account for linguistic factors and enhance its applicability to our context, we utilized a Portuguese-language version adapted in 2009 [39]. We employed the Pittsburgh Sleep Quality Index (PSQI), a tool originally developed by Buysse et al. [40], and subsequently validated it for Brazilian Portuguese speakers [41].

#### 2.3.5. Assessment of Nutritional Parameters

The food intake of the volunteers was investigated via the Food Frequency Questionnaire (FFQ). This questionnaire is able to assess the ingestion of nineteen Brazilian food groups (fruits, vegetables, legumes, milk and dairy products, meat, eggs, sausages, fish, seafood, cereal and breads, sweets, candies, soft drinks, juices, artificial juices, snacks, chips, and alcohol). The records were converted to grams or milliliters, as described previously [42]. The average intake of macronutrients and consequently energy intake were quantified using the Dietbox software (©2021 Dietbox—Version 8.5.0; Rio Grande do Sul, Brazil), selecting the Brazilian Table of Food Composition (TACO) as the basis for the food composition analysis.

### 2.4. Ethical Considerations

All items included in the informed consent form were described in detail to the participants before the start of the questionnaire. Additionally, participants were informed that their data would be handled anonymously, without any personal identification. The Google Forms survey used did not include a field for participants’ names, ensuring their anonymity. This study was approved by the Research Ethics Committee of the University of São Francisco-BR (50748921.9.0000.5514 approved on 27 August 2021). All experiments were conducted in accordance with the relevant guidelines and followed the Declaration of Helsinki. Individuals provided informed consent to participate in this study.

### 2.5. Complex Networks Model and Statistical Analysis

The weighted complex network model was adopted [43,44,45]. Additionally, we directed the network by considering the results from Beck’s Depression Inventory (regarded as the target node). To construct the network, we adopted connections based on significantly correlated variables, with the correlation method determined by the data’s normality (i.e., Pearson or Spearman).

To highlight the most important nodes in relation to the target node, eigenvector analysis was employed. Each edge was assigned a weight based on its proximity to the target node; edges directly connected to the target node were weighted according to their respective correlation coefficients. Second-degree connections to the node of interest were assigned a weight equivalent to 0.500 of the correlation coefficient, while third-degree and fourth-degree connections were assigned weights of 0.250 and 0.125, respectively. In this method, weights reflect the strength of the connections, and the centrality of a node is determined by the centrality of its neighbors through eigenvector centrality analysis.

All centrality analyses were performed in a Python environment (version 3.9.3), specifically developed for this study, utilizing the NetworkX 2.5 library [46]. The Shapiro–Wilk test was employed to verify the normality of the data, and 95.0% confidence intervals were calculated for the mean. For sex comparisons, the Mann–Whitney test for independent samples was used for the analysis of nonparametric variables, while the Kruskal–Wallis test was applied for the comparison of means across groups (G1, G2, G3, and G4). For the scenario involving age groups, outcomes from individuals were categorized as G1 (18–28 years old), G2 (29–39 years), G3 (40–50 years), and G4 (≥51 years). Internal consistency for all dimensions of the questionnaires was assessed using Cronbach’s alpha values, calculated with Statistica 7.0 software (Statsoft Inc., Tulsa, OK, USA).

## 3. Results

General descriptive results of the target node (Beck’s Depression Inventory) as well as physical, psychological, sleep, and nutritional parameters are shown in Table 2. The corresponding 95% confidence intervals are also shown in Table 2. Cronbach’s alpha values for each dimension of the questionnaires are provided in the Supplementary Materials Tables. For the POMSs scales, Cronbach’s alpha values are as follows: tension = 0.72, depression = 0.94, hostility = 0.87, vigor = 0.90, fatigue = 0.88, and confusion = 0.61. The Beck Depression Inventory demonstrated good internal consistency, with Cronbach’s alpha of approximately 0.87. The Epworth Sleepiness Scale exhibited acceptable internal consistency, with Cronbach’s alpha of 0.73 for its eight items. Likewise, the Pittsburgh Sleep Quality Index (PSQI) showed moderate internal consistency, with Cronbach’s alpha of 0.74 based on all of its items.

**Figure 2:**
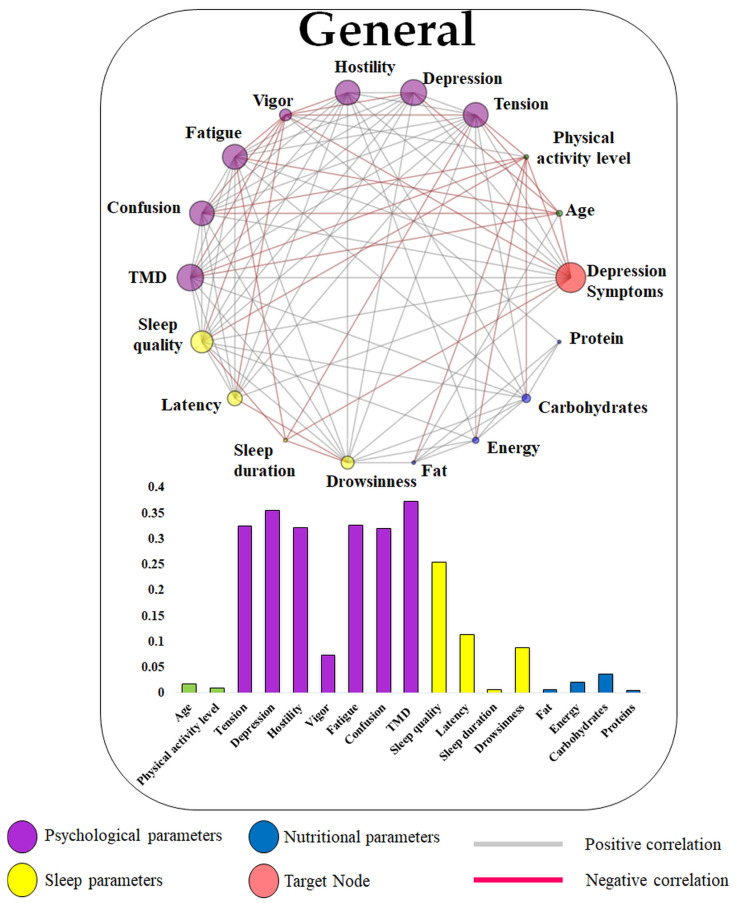
General complex network. The data were not stratified by sex or age, which could provide deeper insights into the findings across the full cohort of 411 participants. The bar graphs represent the eigenvector centrality values of each variable in relation to the target node (depression), where a larger node circumference corresponds to a higher eigenvector centrality value.

**Figure 3:**
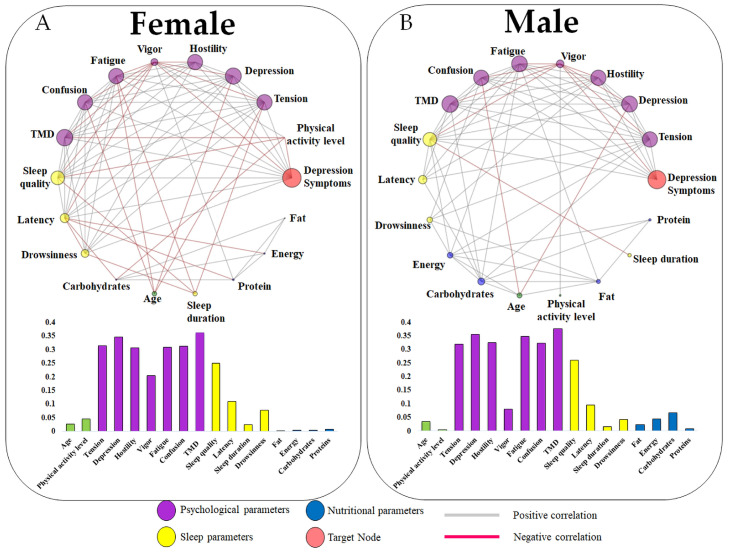
Target complex networks stratified by sex, with females depicted in panel (**A**) and males in panel (**B**). The bar graphs represent the eigenvector centrality values of each variable in relation to the target node (depression), where a larger node circumference corresponds to a higher eigenvector centrality value.

Figure 2 presents the general network. In this analysis, the psychological parameters (except vigor) appeared as the most influential nodes in relation to depressive symptoms. Subsequently, sleep quality was identified as the most significant variable among the sleep parameters. Within nutritional profiles, carbohydrates were notably highlighted compared to proteins, fats, and energy; however, these results, along with age and physical activity level, exhibited less influence on depressive symptoms. A similar profile to the general complex network was observed for the results stratified by sex (Figure 3). For both males and females, the most influential nodes were the same. However, the vigor presented a higher absolute eigenvector value for females (0.203) when compared to males (0.081). Conversely, the nutritional results presented greater importance within the network for males than for females, although both cases showed a low influence on depression symptoms.

Table 3 shows comparisons between male and female individuals. Significant differences were reported regarding all psychological parameters. In comparison to males, females showed higher values of tension, depression, hostility, fatigue, confusion, and TMD. In addition, females showed higher values of Beck’s Depression Inventory (target node in the present study) than males. Conversely, vigor was significantly lower in females compared to males. No differences were found for physical, sleep, and nutritional parameters.

Table 4 presents the comparisons among age groups (G1, G2, G3, and G4). The Kruskal–Wallis test revealed a significant effect on depression, confusion, drowsiness, protein, and fat consumption. Multiple comparison tests indicated that G1 exhibited higher scores for depression and confusion than G3. Additionally, G1 showed greater levels of drowsiness compared to G2. Furthermore, G3 demonstrated higher fat consumption than G1 and also exhibited greater protein intake than G2.

**Figure 4:**
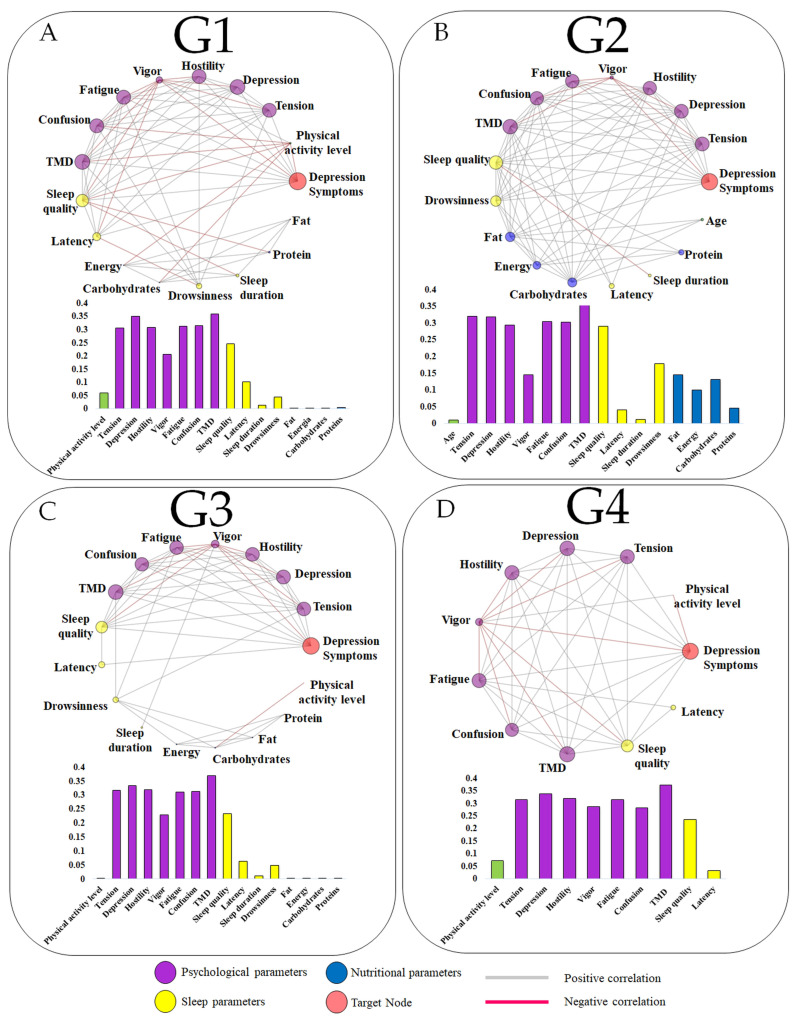
Target networks stratified by age groups: G1 (18–28 years old—panel (**A**)), G2 (29–39 years—panel (**B**)), G3 (40–50 years—panel (**C**)), and G4 (≥51 years—panel (**D**)). The bar graphs represent the eigenvector centrality values of each variable in relation to the target node (depression), where a larger node circumference corresponds to a higher eigenvector centrality value.

The network stratified by age is presented in Figure 4. Like other networks, psychological parameters emerged as the most relevant nodes in all age categories. However, a distinct scenario was observed for G2, where nutritional parameters exerted a greater influence on depressive symptoms compared to the other age groups, as well as the general network and those stratified by sex. Notably, no relationship between nutritional data and the other variables was found for G4, resulting in the absence of these data in the network.

## 4. Discussion

### 4.1. Complex Networks in Diverse Fields

The use of complex networks has become a widely adopted method across various fields for addressing specific problems [47,48,49,50,51,52]. In this context, such analyses serve as a systematic approach and present a promising alternative for investigating the characteristics of different types of interpretations from a macroscopic perspective [53,54,55]. It is important to note that the complex networks model has been applied in diverse areas, including social engagement, topological properties, community health intervention programs, and political analysis [47,48,49,50,51,52,56]. Complex networks have emerged as a powerful framework for studying and understanding the intricate relationships that govern various systems, ranging from social interactions to biological processes and technological infrastructures. A complex network is defined as a collection of interconnected entities, represented as nodes, with edges denoting the relationships or interactions between these nodes. These networks exhibit non-trivial topological features, such as the presence of highly connected hubs, the formation of communities, and small-world properties, which are crucial in shaping their behaviors and functions [57].

### 4.2. The Use of Network Analysis in Depression Studies

Complex networks hold considerable interest for biological scientists; however, their application in the context of depression remains limited. To the best of our knowledge, there are few studies that have effectively utilized network analysis to associate various factors related to depression. For this reason, we will discuss the strengths and weaknesses of some studies to facilitate comparisons and highlight the innovations in our research. Li et al. [25] identified self-hatred as a central symptom of depression in a substantial sample of school-aged students (N = 2,514). The researchers assessed depressive symptoms using the 27-item Child Depression Inventory and employed various centrality indices, including strength, closeness, betweenness, and expected influence, which is certainly the strongest aspect of this study. In a similar way, there is a growing body of research that applies network analysis to understand depression in adolescents and younger students [58,59,60,61,62,63,64]. However, many of these studies have focused primarily on depressive and anxiety symptoms, resulting in a significant gap in the consideration of lifestyle factors, including physical activity and nutrition. In contrast, our study addresses this gap by explicitly incorporating these elements, emphasizing the importance of examining both psychological parameters and lifestyle factors to understand their combined impact on depression.

Wang et al. [23] explored connections between lifestyles and depression–anxiety symptoms, and a notable strength of this study was the substantial sample size of 13,768 residents from Guangdong province (China). Another key point to highlight is that Wang et al. [23] examined current tobacco and alcohol consumption, both of which were positively associated with suicidal thoughts and irritability. In a particularly interesting strategy, Anderson et al. [24] not only focused on lifestyle but also included social determinants such as socioeconomic status, social interaction, and racial status in a large sample of U.S. adults. Given the importance of tobacco and alcohol consumption, along with social determinants, in the context of depression, it is evident that our study was lacking in data regarding these factors compared to the research conducted by Wang et al. [23] and Anderson et al. [24]. Despite this limitation, our evaluation of physical activity stands out as a strength. Wang et al. [23] assessed physical exercise frequency through a simple question “How often did you work out in the past year?”, which limited the potential for a more in-depth exploration. Anderson et al. [24] employed a more refined assessment by evaluating engagement in moderate and vigorous physical activity. In contrast to these two studies, we used a questionnaire that considers not only moderate and vigorous activities but also any walking activities, as well as sedentary behavior by measuring the time spent sitting during workdays and weekends. Regarding nutritional data, Wang et al. [23] was the only study that inquired about participants’ habitual dietary rhythm. In contrast, our study measured dietary intake with greater detail by assessing average macronutrient and micronutrient consumption, along with daily energy intake.

Considering the existing literature, our primary novelty lies in constructing networks based on specific population segments, examining differences by sex and stratifying participants by age into four distinct categories. While our strategy can reveal unique patterns and relationships that may be obscured in broader networks, a limitation of our study is the absence of associations among variables over an extended period through within-person analysis. Such analysis was effectively carried out by Anderson et al. [24], who assessed data over nearly two decades (1995–2014). While the network can easily identify the most prominent variables, this is not its sole objective. It is undeniable that networks are useful for identifying central variables; by doing so, researchers and practitioners can design more effective interventions. For example, if TMD emerges as a central factor, interventions can specifically target strategies to enhance mental health. It is important to note that our analysis aims to use a model that mathematically assembles a system of variables. We opted to customize the eigenvector analysis by introducing weights based on the proximity of variables to the target variable. This approach offers some advantages: (1) It is more suitable for systems consisting of mixed-class biological variables, as it reduces the influence of covariance among variables of the same class. (2) Our weighting method enhances our understanding of both direct and indirect relationships within the network, allowing for a more nuanced analysis of interactions between variables. To illustrate this, we will now explore an example using a weight-adjusted network model. In our approach, edges directly connected to the target node are assigned a weight of 1, reflecting the correlation coefficient itself. For example, the direct correlation between the target node (the score from Beck’s Depression Inventory) and sleep quality (r = 0.52) illustrates this principle. Any variable correlated with sleep quality will also be highlighted in the network. To further clarify, a second-degree connection between sleep quality and carbohydrate consumption (r = 0.13) would be adjusted to a lower weight, calculated as 0.13 × 0.5 = 0.065. Therefore, in global interpretation, the positive correlation between sleep quality and the target node suggests that individuals with higher levels of depression tend to have reduced sleep quality. Meanwhile, the indirect relationship (the second-degree) between sleep quality and carbohydrate consumption could imply that dietary habits, specifically carbohydrate intake, may have a modest influence on sleep quality, which in turn could be involved in depression.

### 4.3. General Network

The findings, analyzed using the eigenvector metric, revealed significant interconnections among nodes, illustrating relationships between depression symptoms and psychological parameters such as hostility, tension, fatigue, TMD, and vigor. This is consistent with the literature that has shown the relationship between mood swings and symptoms of depression [3,15]. Notably, vigor remained consistently low across all parameters (general, age, and sex), whereas the other psychological elements—tension, depression, hostility, fatigue, confusion, and TMD—remained elevated.

In line with the literature, our data appear to indicate that the occurrence and/or worsening of depression is influenced by poor sleep quality [18,19,65], mood disturbances [3,66,67], and sedentary behavior [66,68]. These factors can trigger a pro-inflammatory state in the body, potentially leading to inflammation [69,70,71] and worsening depressive symptoms [72,73]. It is important to note that metabolic diseases are associated with depression, which increases the risk of both disability and mortality [67,74].

Our results indicated a positive correlation between sleep quality and depressive symptoms (target node). While sleep-related aspects are already documented in the scientific literature [17,18,65], it is crucial to highlight that this study is pioneering in illustrating the interconnections between depressive symptoms and various parameters within a single network. The positive correlation between sleep quality and depressive symptoms likely arises from the scoring system used, where a higher score reflects poorer sleep quality. Thus, although the correlation value is positive, it actually signifies a decline in sleep quality in relation to the presence of depressive symptoms.

Although the correlation value for physical activity was not particularly strong, it reveals an interesting interaction with other parameters such as fatigue, tension, vigor, carbohydrate and fat consumption, and aspects of sleep profile, highlighting the multiple correlations within the complex network. Engaging in physical activity directly influences dietary habits, which can, in turn, affect sleep quality, psychological factors, and, ultimately, the emergence of depressive symptoms [6,75].

### 4.4. Influence of Sexes on the Network

Although nutritional aspects have minimal influence on the network, they appear to be slightly more relevant in the male network compared to the female network (Figure 3B). Dietary changes can impact the gut microbiome, which affects mood and plays a complex role in the bidirectional interaction between brain function, inflammation, and neurotransmitters [76,77]. Stress, a significant predisposing factor for depression [78], is also linked to dietary habits, including the excessive consumption of sugar and processed foods, which are correlated with heightened depressive symptoms [9,79].

Regarding sleep profile, sleep quality emerges as a key factor for both females and males, ranking among the most prominent variable in all networks. The nodes indicate that low sleep quality adversely affects other aspects, as evidenced by the interconnections with physical activity, sleep duration, and vigor. Curiously, physical activity appears to be more pronounced in females, suggesting a negative correlation with sleep quality, TMD, and carbohydrate consumption. The existing literature suggests that females are at a higher risk of developing depressive symptoms [31,80] and tend to have a greater incidence of eating disorders, often using food as an emotional regulator [81].

### 4.5. Influence of Age on the Network

Regarding the network stratified by age, the nodes reveal variations based on nutritional parameters. As shown in Figure 4, these parameters are more pronounced in G2, which includes individuals aged 29 to 39 years, indicating that those in this age category may experience stronger links between dietary factors and depressive symptoms. Across all age categories, sleep and psychological parameters emerged as the most relevant, underscoring their critical role in the likelihood of developing depressive symptoms. Furthermore, an additional interpretation of the network analysis indicates that sleep quality consistently stands out as a predominant factor compared to other sleep-related parameters across all age categories.

Several studies in the literature suggest that variations in sleep can lead to mood swings, which in turn may increase depressive symptoms [2,17,65], corroborating the findings in our work. In addition to these highlights, other age-related divergences were noted in the arrangement of variables within the network, but we are currently unable to further our explanations. New studies must be conducted to better elucidate this issue.

### 4.6. Limitations and Future Perspectives

Every study has its limitations, and ours is no exception. We collected variables through questionnaires, which limited our ability to use equipment for direct measurement and monitoring of participants. While self-reported data can introduce potential biases, we utilized validated questionnaires that are sufficiently reliable to support a trustworthy analysis. All questionnaires were specifically validated for the Portuguese language. It is evident that improvements will need to be made to mitigate the limitations of experiments of this nature. Future initiatives should aim to obtain results from different populations, including other countries with diverse socioeconomic backgrounds, thereby enhancing and broadening the reliability of data. Increasing the sample size will be crucial for gaining a deeper understanding of all possible variables in the context of depression. Although our study had a relatively moderate sample size, it may have identified probable behavioral patterns that could be valuable for future research.

To maintain the accuracy and caution of our interpretations, we must emphasize the correlational nature of our findings. Although we identified relationships between variables, it is essential to clarify that these correlations do not imply causation. Therefore, we cannot assert that one variable influences another or generalizes our results beyond the specific context of our sample. Recognizing that complex networks may present an unfamiliar blueprint for some researchers, a summary of the main results is also available through a video abstract S1 (Supplementary Materials). This approach has been suggested to enhance visibility and scientific communication [82].

Integrative data on non-pharmacological treatments remain scarce in the literature. Considering that factors such as physical activity, sleep profile, and nutritional aspects are strongly recommended to mitigate the progression of depression [4,83], there is a significant open avenue for research on these variables collectively and their holistic impact on depression. This area is likely to gain prominence in the coming years.

## 5. Conclusions

In the context of depression, this study is the first to construct target networks using eigenvector analysis across different subgroups, considering not only general aspects (incorporating all data) but also accounting for sex differences (female vs. male) and stratifying age into four categories. Interesting and significant interconnections among physical activity, dietary patterns, sleep profiles, and psychological aspects were found. Specifically, the highest-ranked variables identified were psychological parameters, including tension, depression, hostility, fatigue, and confusion (assessed by the Profile of Mood States), as well as sleep quality (measured by the Pittsburgh Sleep Quality Index) for all subgroups. Despite the prominence of mood states and sleep quality, other variables highlighted specific nuances in certain contexts. Notably, stronger connections between nutritional parameters and depressive symptoms were observed within the male network and among individuals aged 29–39 years (G2 category). Another notable finding is that, compared to other sleep-related parameters (sleep latency, sleep duration, and drowsiness), sleep quality consistently stands out as a predominant factor, emphasizing the need for researchers to prioritize it in their studies. Given its multifactorial nature, it is essential for future studies to integrate additional variables within the network, providing stronger support for future multidisciplinary approaches.

## Figures and Tables

**Table 1:** Frequency distribution of the sample group according to sex and age.

Characteristic	Categories	N	%
**All**	**General**	411	100%
**Sex**	**Male**	89	21.7%
	**Female**	322	78.3%
**Age**	**G1 (18–28 years)**	241	58.6%
	**G2 (29–39 years)**	70	17.0%
	**G3 (40–50 years)**	60	14.6%
	**G4 (≥51 years)**	40	9.7%

**Table 2:** General descriptive results for all participants (N = 411).

		M	SD	Min–Max	Confidence Interval
**Target** **Node**	Beck’s Depression Inventory	11.9	8.5	0–46	11.1–12.7
**Physical Parameters**	Age (years)	30.7	12.4	18–74	29.5–31.9
	Physical activity level (MET-minutes/week)	2191.9	2223.6	0–17,200.0	1973.5–2410.1
**Psychological Parameters**	Tension (score)	11.3	5.5	0–24	10.7–11.8
	Depression (score)	12.2	11.3	0–45	11.0–13.3
	Hostility (score)	6.5	5.2	0–24	6.0–7.0
	Vigor (score)	10.5	5.5	0–24	9.9–11.0
	Fatigue (score)	10.0	6.2	0–24	9.3–10.5
	Confusion (score)	8.6	4.5	0–22	8.1–9.0
	TMD (score)	137.6	32.2	17–232	134.4–140.8
**Sleep Parameters**	Sleep quality (score)	6.2	2.9	0–16	5.9–6.5
	Sleep latency (min)	26.8	25.6	0–210.0	24.2–29.2
	Sleep duration (h)	7.8	1.4	3.1–13.0	7.6–7.9
	Drowsiness (score)	9.0	4.7	0–22	8.4–9.4
**Nutritional Parameters**	Energy (kcal)	3187.0	1137.9	993.3–8176.4	3076.6–3297.2
	Carbohydrates (g)	348.0	140.6	69.2–937.2	334.3–361.6
	Proteins (g)	157.3	52.5	31.8–343.8	152.2–162.4
	Fat (g)	132.0	50.3	30.7–359.8	127.0–136.8

Data are in mean (M) and standard deviation (SD). Column labeled “Min–Max” represents, respectively, the minimum and the maximum value observed in the experiment.

**Table 3:** Characteristics of individuals according to sex.

		Female	Male	Between-Group Comparisons
**Target** **Node**	Beck’s Depression Inventory	12.6 ± 8.2	9.8 ± 9.1	**Z = 3.84, *p* < 0.001**
**Physical** **Parameters**	Age (years)	31.1 ± 12.7	29.4 ± 11.3	Z = 0.67, *p* = 0.498
	Physical activity level (MET-minutes/week)	2133.8 ± 2311.5	2395.6 ± 1880.6	Z = 1.80, *p* = 0.071
**Psychological Parameters**	Tension (score)	11.9 ± 5.4	9.0 ± 4.9	**Z = 4.31, *p* < 0.001**
	Depression (score)	12.7 ± 11.3	10.4 ± 11.4	**Z = 2.22, *p* = 0.025**
	Hostility (score)	6.8 ± 5.3	5.5 ± 5.0	**Z = 2.53, *p* = 0.011**
	Vigor (score)	10.0 ± 5.4	12.5 ± 5.4	**Z = 3.78, *p* < 0.001**
	Fatigue (score)	10.7 ± 6.2	7.6 ± 5.8	**Z = 4.26, *p* < 0.001**
	Confusion (score)	8.9 ± 4.4	7.3 ± 4.6	**Z = 3.26, *p* = 0.001**
	TMD (score)	140.6 ± 31.9	127.3 ± 31.3	**Z = 3.82, *p* < 0.001**
**Sleep** **Parameters**	Sleep quality (score)	6.3 ± 2.9	5.9 ± 2.7	Z = 1.12, *p* = 0.262
	Sleep latency (min)	27.3 ± 26.1	25.2 ± 23.8	Z = 0.88, *p* = 0.374
	Sleep duration (h)	8.0 ± 1.1	8.0 ± 1.4	Z = 1.37, *p* = 0.168
	Drowsiness (score)	9.0 ± 4.9	8.7 ± 4.2	Z = 0.31, *p* = 0.756
**Nutritional** **Parameters**	Energy (kcal)	3190.9 ± 1170.6	3172.7 ± 1016.9	Z = 0.11, *p* = 0.906
	Carbohydrates (g)	350.2 ± 144.3	340.1 ± 126.6	Z = 0.56, *p* = 0.571
	Proteins (g)	155.6 ± 53.3	163.7 ± 49.2	Z = 1.40, *p* = 0.160
	Fat (g)	132.4 ± 51.7	130.3 ± 45.3	Z = 0.28, *p* = 0.773

Data are in mean and SD. As data were not normally distributed, the Mann–Whitney test was used for comparison of two means.

**Table 4:** Characteristics of individuals according to age.

		G1 (18–28 Years)	G2 (29–39 Years)	G3 (40–50 Years)	G4 (≥51 Years)	Between-Group Comparisons
**Target** **Node**	Beck’s Depression Inventory	13.1 ± 9.2	10.0 ± 7.2	10.6 ± 7.3	10.6 ± 6.6	H = 7.92, *p* = 0.047
**Physical** **Parameters**	Age (years)	22.0 ± 3.0	33.7 ± 3.2 **a**	44.5 ± 2.9 **a b**	57.2 ± 5.6 **a b**	**H = 324.46, *p* < 0.001**
	Physical activity level (MET-minutes/week)	2220.5± 2326.5	2249.8± 2289.9	2282.1± 2173.7	1770.9± 1400.6	H = 0.50, *p* = 0.916
**Psychological Parameters**	Tension (score)	11.7 ± 5.2	11.2 ± 6.0	10.7 ± 5.5	9.8 ± 5.7	H = 5.91, *p* = 0.116
	Depression (score)	13.6 ± 11.6	11.1 ± 11.2	9.3 ± 10.3 **a**	10.3 ± 10.5	**H = 11.50, *p* = 0.009**
	Hostility (score)	6.7 ± 5.2	6.6 ± 5.7	6.4 ± 5.1	5.8 ± 5.3	H = 1.69, *p* = 0.637
	Vigor (score)	10.6 ± 5.4	10.4 ± 5.0	10.8 ± 5.9	10.0 ± 6.1	H = 0.45, *p* = 0.929
	Fatigue (score)	10.4 ± 6.1	10.0 ± 6.5	9.5 ± 6.4	8.0 ± 6.0	H = 6.24, *p* = 0.100
	Confusion (score)	9.4 ± 4.6	8.1 ± 4.5	6.9 ± 4.1 **a**	7.3 ± 3.5	**H = 17.58, *p* < 0.001**
	TMD (score)	140.4 ± 32.3	136.6 ± 32.8	131.9 ± 31.0	131.2 ± 31.2	H = 6.30, *p* = 0.097
**Sleep** **Parameters**	Sleep quality (score)	6.2 ± 2.9	6.2 ± 2.9	6.2 ± 2.7	6.4 ± 3.1	H = 0.09, *p* = 0.992
	Sleep latency (min)	27.4 ± 26.0	26.5 ± 20.0	21.7 ± 19.3	31.3 ± 36.6	H = 4.07, *p* = 0.253
	Sleep duration (h)	7.9 ± 1.5	7.6 ± 1.4	7.5 ± 1.1	8.0 ± 1.2	H = 7.74, *p* = 0.051
	Drowsiness (score)	9.5 ± 4.4	7.6 ± 4.6 **a**	8.6 ± 5.4	8.7 ± 5.1	**H = 10.16, *p* = 0.017**
**Nutritional** **Parameters**	Energy (kcal)	3139.5 ± 1105.5	3082.6 ± 1289.3	3489.9 ± 1112.4	3201.1± 1050.5	H = 7.11, *p* = 0.068
	Carbohydrates (g)	347.8 ± 138.9	332.7 ± 158.4	374.1 ± 133.4	337.0 ± 126.8	H = 4.61, *p* = 0.201
	Proteins (g)	154.7 ± 51.9	152.3 ± 58.6	171.5 ± 49.3 **b**	160.7 ± 47.7	**H = 7.88, *p* = 0.048**
	Fat (g)	127.8 ± 48.7	129.4 ± 55.5	148.2 ± 50.9 **a**	137.2 ± 46.0	**H = 10.03, *p* = 0.018**

Data are in mean and SD. When Kruskal–Wallis test showed a significant difference between groups, a multiple comparison test was used to determine which pairs of means were significantly different. Statistical analysis: a, b: significant difference (*p* < 0.05) in relation to G1 and G1, respectively.

## Data Availability

The datasets used and/or analyzed during the current study are available from the corresponding author on reasonable request.

## Supplementary Materials

The following supporting information can be downloaded at: https://www.mdpi.com/article/10.3390/jcm13226743/s1. To address the extensive correlations, we included the correlation matrices for network construction as part of the Supplementary Materials. Separate analyses for each subgroup (general, male, female, G1, G2, G3, and G4) resulted in twenty-one Excel files: seven for correlation coefficients (r), seven for *p*-values, and seven for eigenvector results, allowing for a thorough review of our findings. Table S1: The internal consistency analysis resulted in four pdf files, containing Cronbach’s alpha values for the Beck Depression Inventory; Table S2: Profile of Mood States; Table S3: Pittsburgh Sleep Quality Index; Table S4: Epworth Sleepiness Scale; Video S1: Using the complex network model to associate nutritional, psychological, and physical parameters and aspects of sleep with depression symptoms.
