# Supplementary material for: Using the Complex Network Model to Associate Nutritional, Psychological, and Physical Parameters and Aspects of Sleep with Depression Symptoms
Source: J Clin Med. 2024 Nov 9;13(22):6743. doi: 10.3390/jcm13226743 (PMC11594319; doi:10.3390/jcm13226743)
Supplement: Supplementary file 1 [file jcm-13-06743-s001.zip › Table S1.pdf]

Table S1. Internal consistency data for the 21 items of the Beck Depression Inventory.

Dimension	Item	Item-total correlation	Cronbach's alpha if item deleted	Cronbach's alpha
Major depression	Sadness	0.61	0.86	0.87
	Pessimism	0.53	0.86	
	Sense of failure	0.58	0.86	
	Lack of satisfaction	0.58	0.86	
	Guilty feelings	0.63	0.86	
	Sense of punishment	0.44	0.87	
	Self-dislike	0.56	0.86	
	Self-accusations	0.59	0.86	
	Suicidal wishes	0.46	0.87	
	Crying spells	0.43	0.87	
	Irritability	0.28	0.87	
	Social withdrawal	0.50	0.86	
	Indecisiveness	0.57	0.86	
	Distortion of body image	0.56	0.86	
	Work inhibition	0.55	0.86	
	Sleep disturbance	0.39	0.87	
	Fatigability	0.57	0.86	
	Loss of appetite	0.38	0.87	
	Weight loss	0.16	0.88	
	Somatic preoccupation	0.35	0.87	
	Loss of libido	0.42	0.87	

The table also presents the correlation values for each item, where low correlations suggest that the item may not align well with the rest of the scale. Additionally, it shows the internal consistency (Coefficient Alpha) of the scale that would result if each item were removed.
