# Supplementary material for: Using the Complex Network Model to Associate Nutritional, Psychological, and Physical Parameters and Aspects of Sleep with Depression Symptoms
Source: J Clin Med. 2024 Nov 9;13(22):6743. doi: 10.3390/jcm13226743 (PMC11594319; doi:10.3390/jcm13226743)
Supplement: Supplementary file 1 [file jcm-13-06743-s001.zip › Table S2.pdf]

Table S2. Internal consistency data for the 42-item version of the POMS and Cronbach's alpha values for its six dimensions

Dimension	Item	Item-total correlation	Cronbach's alpha if item deleted	Cronbach's Alpha in the dimension
**Tension**	Tense	0.56	0.65	0.72
	Restless	0.60	0.64	
	Nervous	0.64	0.63	
	Anxious	0.70	0.61	
	Calm	-0.29	0.86	
	Impatient	0.68	0.62	
**Depression**	Useless	0.75	0.93	0.94
	Worthless	0.75	0.93	
	Miserable	0.70	0.94	
	Useless	0.78	0.93	
	Guilty	0.67	0.94	
	Apathetic	0.68	0.94	
	Sad	0.74	0.93	
	Discouraged	0.78	0.93	
	Alone	0.65	0.94	
	Depressed	0.83	0.93	
	Discouraged	0.76	0.93	
	Unhappy	0.80	0.93	
**Hostility**	Irritated	0.69	0.85	0.87
	Bad-tempered	0.73	0.84	
	Annoyed	0.73	0.84	
	Furious	0.73	0.84	
	Ill-tempered	0.49	0.88	
	Enervated	0.70	0.85	
**Vigor**	Excited	0.77	0.87	0.90
	Active	0.62	0.90	
	Energetic	0.70	0.88	
	Full of life	0.77	0.87	
	Cheerful	0.76	0.87	
	Happy	0.75	0.88	
**Fatigue**	Exhausted	0.78	0.85	0.88
	Fatigued	0.67	0.87	
	Worn out	0.80	0.85	
	Low-energy	0.71	0.86	
	Burned out	0.48	0.90	
	Tired	0.78	0.85	
**Confusion**	Confused	0.52	0.48	0.61
	Disoriented	0.57	0.47	
	Effective	0.11	0.64	
	Competent	0.02	0.68	
	Insecure	0.38	0.54	
	Confused	0.51	0.49	

The table also presents the correlation values for each item, where low correlations suggest that the item may not align well with the rest of the scale. Additionally, it shows the internal consistency (Coefficient Alpha) of the scale that would result if each item were removed
