# Supplementary material for: Using the Complex Network Model to Associate Nutritional, Psychological, and Physical Parameters and Aspects of Sleep with Depression Symptoms
Source: J Clin Med. 2024 Nov 9;13(22):6743. doi: 10.3390/jcm13226743 (PMC11594319; doi:10.3390/jcm13226743)
Supplement: Supplementary file 1 [file jcm-13-06743-s001.zip › Table S3.pdf]

**Table S3.** Internal consistency data for the items of the Pittsburgh Sleep Quality Index (PSQI). This questionnaire encompasses the following components: subjective sleep quality (C1), sleep latency (C2), sleep duration (C3), habitual sleep efficiency (C4), sleep disturbances (C5), use of sleeping medication (C6), and daytime dysfunction (C7). The table presents the overall Cronbach's alpha for the PSQI, calculated based on all items.

| Dimension | Item | Item-total correlation | Cronbach's alpha if item deleted | Cronbach's alpha in the dimension | Cronbach's alpha |
|---|---|---|---|---|---|
| **Subjective sleep quality** | **Q#6** | Too few items | | | 0.74 |
| **Sleep latency** | **Q#2** | Too few items | | | |
| | **Q#5a** | | | | |
| **Sleep duration** | **Q#4** | Too few items | | | |
| **Habitual sleep efficiency** | Percentage (%) analysis of the data on the number of hours slept and the number of hours spent in bed | | | | |
| **Sleep disturbances** | **Q#5b** | 0.39 | 0.63 | 0.67 | |
| | **Q#5c** | 0.33 | 0.64 | | |
| | **Q#5d** | 0.33 | 0.64 | | |
| | **Q#5e** | 0.26 | 0.66 | | |
| | **Q#5f** | 0.35 | 0.64 | | |
| | **Q#5g** | 0.35 | 0.64 | | |
| | **Q#5h** | 0.27 | 0.66 | | |
| | **Q#5i** | 0.43 | 0.62 | | |
| | **Q#5j** | 0.38 | 0.63 | | |
| **Use of sleeping medication** | **Q#7** | Too few items | | | |
| **Daytime dysfunction** | **Q#8** | Too few items | | | |
| | **Q#9** | | | | |

The table also presents the correlation values for each item, where low correlations suggest that the item may not align well with the rest of the scale. Additionally, it shows the internal consistency (Coefficient Alpha) of the scale that would result if each item were removed
