# Supplementary material for: Using the Complex Network Model to Associate Nutritional, Psychological, and Physical Parameters and Aspects of Sleep with Depression Symptoms
Source: J Clin Med. 2024 Nov 9;13(22):6743. doi: 10.3390/jcm13226743 (PMC11594319; doi:10.3390/jcm13226743)
Supplement: Supplementary file 1 [file jcm-13-06743-s001.zip › Table S4.pdf]

**Table S4.** Internal consistency data for the 8 items of the Epworth Sleepiness Scale, which assess the tendency to doze.

| Dimension | Item | Item-total correlation | Cronbach's alpha if item deleted | Cronbach's alpha |
|---|---|---|---|---|
| **Chance of Dozing** | Sitting and reading | 0.45 | 0.70 | 0.73 |
| | Watching TV | 0.36 | 0.71 | |
| | Sitting inactive in a public place | 0.48 | 0.69 | |
| | As a passenger in a car for an hour without a break | 0.47 | 0.69 | |
| | Lying down to rest in the afternoon when circumstances permit | 0.39 | 0.71 | |
| | Sitting and talking to someone | 0.38 | 0.71 | |
| | Sitting quietly after a lunch without alcohol | 0.42 | 0.70 | |
| | In a car, while stopped for a few minutes in traffic | 0.43 | 0.70 | |

The table also presents the correlation values for each item, where low correlations suggest that the item may not align well with the rest of the scale. Additionally, it shows the internal consistency (Coefficient Alpha) of the scale that would result if each item were removed
